# Small extracellular vesicles derived from mesenchymal stem cell facilitate functional recovery in spinal cord injury by activating neural stem cells *via* the ERK1/2 pathway

**DOI:** 10.3389/fncel.2022.954597

**Published:** 2022-08-29

**Authors:** Xinyuan Hu, Zhong Liu, Xinru Zhou, Qian Jin, Wenrong Xu, Xiao Zhai, Qiang Fu, Hui Qian

**Affiliations:** ^1^Key Laboratory of Laboratory Medicine of Jiangsu Province, School of Medicine, Jiangsu University, Zhenjiang, China; ^2^Department of Clinical Laboratory, Qingdao Municipal Hospital, Qingdao, China; ^3^Department of Orthopedics, Shanghai General Hospital, Shanghai Jiao Tong University School of Medicine, Shanghai, China; ^4^Department of Laboratory Diagnostics, Changhai Hospital, Naval Medical University, Shanghai, China; ^5^Department of Orthopedics, Changhai Hospital, Naval Medical University, Shanghai, China; ^6^NHC Key Laboratory of Medical Embryogenesis and Developmental Molecular Biology, Shanghai Key Laboratory of Embryo and Reproduction Engineering, Shanghai, China

**Keywords:** small extracellular vesicles, spinal cord injury, anti-inflammation, neurogenesis, neural stem cells, ERK1/2 pathway

## Abstract

Spinal cord injury (SCI) causes severe neurological dysfunction leading to a devastating disease of the central nervous system that is associated with high rates of disability and mortality. Small extracellular vesicles (sEVs) derived from human umbilical cord mesenchymal stem cells (hucMSC-sEVs) have been explored as a promising strategy for treating SCI. In this study, we investigated the therapeutic effects of the intralesional administration of hucMSC-sEVs after SCI and determined the potential mechanisms of successful repair by hucMSC-sEVs. *In vivo*, we established the rat model of SCI. The Basso, Beattie, Bresnahan (BBB) scores showed that hucMSC-sEVs dramatically promoted the recovery of spinal cord function. The results of the hematoxylin–eosin (HE) staining, Enzyme-Linked Immunosorbent Assay (ELISA), and immunohistochemistry showed that hucMSC-sEVs inhibited inflammation and the activation of glia, and promoted neurogenesis. Furthermore, we studied the effect of hucMSC-sEVs on neural stem cells(NSCs) *in vitro*. We found that hucMSC-sEVs did not improve the migration ability of NSCs, but promoted NSCs to proliferate and differentiate *via* the ERK1/2 signaling pathway. Collectively, these findings suggested that hucMSC-sEVs promoted the functional recovery of SCI by activating neural stem cells *via* the ERK1/2 pathway and may provide a new perspective and therapeutic strategy for the clinical application of hucMSC-sEVs in SCI treatment.

## Introduction

The inability of the spinal cord to regenerate after injury is predominantly associated with the death of neurons along with the formation of a cavity and an inhibitory microenvironment. At present, there are no effective treatments that can cure these neurological deficits (Haus et al., 2016; Andrzejewska et al., 2021). However, a significant progress has been achieved in the regeneration of the central and peripheral nervous system and stem cell-based transplantation treatment has been considered as a potential means of curing spinal cord injury (SCI) in the near future (Lin et al., 2018; Ahuja et al., 2020).

Small extracellular vesicles (sEVs) are lipid bilayer nanoparticles secreted by multiple cells and provide a potential model for intercellular communication that transfers a range of bioactive cargoes both locally and distally (Negahdaripour et al., 2021). Recent studies have demonstrated that the therapeutic potential of mesenchymal stem cells (MSCs) can be mainly attributed to their secretion of numerous paracrine factors, particularly sEVs. For instance, sEVs obtained from stem cells derived from human adipose effectively protected cartilage from degeneration and attenuated the progression of osteoarthritis by regulating immune reactivity (Woo et al., 2020). Moreover, sEVs secreted by MSCs derived from human-induced pluripotent stem cells have been shown to protect against ischemic stroke and enhance angiogenesis potentially by the inhibition of autophagy (Xia et al., 2020). Furthermore, it has also been reported that human umbilical cord mesenchymal stem cell-derived small extracellular vesicles (hucMSC-sEVs) alleviated bronchopulmonary dysplasia by improving alveolarization and promoting angiogenesis through the PTEN/Akt signaling pathway (You et al., 2020). Even though the protective potential of sEVs derived from MSCs has been widely validated and the molecular mechanisms have been preliminarily elucidated, we are still in the preliminary stages of understanding the full capability of these sEVs. The purpose of the present study was to investigate the therapeutic role of hucMSC-sEVs in SCI and explore the underlying mechanism from a novel perspective. Our results indicated that hucMSC-sEVs promoted functional recovery of SCI by promoting endogenous neural stem cells to proliferate and differentiate *via* the activation of the ERK1/2 pathway rather than by inducing neural stem cell migration into the lesion site and by enhancing the outgrowth of axonal fibers through the lesion site.

## Materials and methods

### Cell culture

Mesenchymal stem cells derived from the human umbilical cord (hucMSCs) were isolated and identified as previously described (Chandravanshi and Bhonde, 2018). HucMSCs were cultured in a 10% serum α-Minimum Essential Medium (MEM) medium. Dorsal root ganglia (DRGs) were collected from newborn Sprague–Dawley rats and cultured in a neurobasal medium containing 2% (v/v) B27, 2.5 g/L glucose, 10 ng/L NGF, and 2 mmol/L (+)-Glutamine. HucMSCs and DRGs were both cultured at 37°C with 5% CO_2_. Neural stem cells(NSCs) were isolated from the foreheads of neonatal Sprague–Dawley rats, as described previously (Ahlenius and Kokaia, 2010). NSCs were incubated with 2% B27, EGF (20 ng/ml), and bFGF (20 ng/ml) daily in serum-free DMEM/F12 medium at 37°C with 5% CO_2_. After 3 days of culture, neurospheres were formed and plated into 24-well plates for detection.

### Isolation and identification of hucMSC-sEVs

Small extracellular vesicles derived from hucMSCs were isolated and purified as described previously (Wu et al., 2021). Cell supernatants were collected and centrifuged at 1,500 × *g* for 20 mins to remove cell debris and then centrifuged at 10,000 × *g* for 30 min. Next, the sEVs were concentrated with a 100 kDa molecular weight cutoff ultrafiltration membrane (MWCO) (Millipore, Billerica, MA, United States). Subsequently, the concentrated solution from the upper tube was collected for ultracentrifugation at 100,000 g for 70 min at 4°C to pellet the hucMSC-sEVs. Then pellet containing sEVs was resuspended in phosphate-buffered saline (PBS) and centrifuged again at 100,000 g for 70 min. Finally, the pelleted sEVs were washed and gathered from the bottom of the tube with PBS. sEVs were finally filtered with a 0.22 μm pore filter (Millipore, Billerica, Massachusetts, United States) and stored at −80°C. The diameter of the concentrated sEVs was determined by nanoparticle tracking analysis (NTA) (NanoSight, Amesbury, United Kingdom). sEVs were also identified morphologically by transmission electron microscopy (FEI Tecnai 12, Philips, Netherlands). Western blotting was also used to confirm the presence of characteristic markers of sEVs (CD81, HSP70, and Alix).

### Construction of a rat model of spinal cord injury and hucMSC-sEVs treatment

Adult female Sprague–Dawley rats (weighing 220–250 g) were purchased from the Animal Centre of Jiangsu University (Zhenjiang, China) and randomly divided into two groups: SCI + PBS (*n* = 6) and SCI + hucMSC-sEVs (*n* = 6). Anesthesia was induced by 8% chloralhydrate (5 ml/kg) *via* intraperitoneal injection. Laminectomy at the T6-T7 vertebral level was then performed to expose the spinal cord. For the transverse SCI models, the spinal cord was transected, immediately after hemostasis, 50 μl of either PBS or PBS containing hucMSC-sEVs (2 mg total protein, Bicinchoninic acid assay) were slowly injected into the upper and lower sides of the spinal cord transected site with a depth of 0.9 mm using a pulled-glass micropipette. After the solution was absorbed, the musculature and skin were sutured sequentially. All rats received careful post-operative care. Rat models of SCI were subsequently analyzed by micro-CT (Quantum GX, PerkinElmer, United States) to identify the injury site and hucMSC-sEVs treated rats were analyzed by a small animal *in vivo* imaging system (*In Vivo* Xtreme II, Bruker Biospin, United States) to evaluate the long-term presence of the Dir- hucMSC-sEVs at the injury site 1 week after surgery.

### Behavior assessments

Locomotor function was assessed using the Beattie–Beattie–Bresnahan (BBB) locomotor rating scale by evaluators who were blinded to treatment conditions (Basso et al., 1995). All rats were scored 1 day prior to surgery and at weekly intervals until sacrifice at 8 weeks post-SCI.

### Neuroanatomical tracing

A trans-synaptic retrograde tracing technique was used for neuroanatomical tracing. After 4 weeks of surgery, each animal model was anesthetized, and the spinal cord was re-exposed at the level of the injury site. Next, 1 μl of fluorescent-gold solution (20 ng/ml) was injected 5 mm below the injury site using a microsyringe. These rats were housed for one more week; then, the spinal cords were collected and positive neurons were counted in frozen sections created from the tissue on both sides of the SCI. Images were acquired by fluorescence microscopy (BX53; Olympus, Tokyo, Japan).

### Migration assay for neural stem cells *in vitro*

To determine the radial migration of NSCs, a single neural stem cell sphere was collected and transferred into a 24-well plate coated with Matrigel. All cells in each 24-well plate were counterstained with hoechst33342 for 36 h after treatment with hucMSC-sEVs. The furthest distance migrated from the sphere was taken to represent the potential for migration and representative images were acquired by fluorescence microscopy (BX53; Olympus, Tokyo, Japan).

### Immunofluorescence staining

Neural stem cells and DRGs were fixed with 4% PFA solution at 4°C overnight. After rinsing three times with cold PBS, neural stem cells were incubated with 5% BSA (bovine serum album) solution and 0.3% triton solution at 37°C for 30 min and with the following primary antibodies overnight: anti-Gap-43 (1:200, CST, United States), anti-PSD95 (1:200, BOSTER, CHINA), anti-GFAP (1:200, Bioworld, United States), and anti-β-III-tubulin (1:100, abmgood, Canada). After rinsing in PBS, neural stem cells were incubated with a cy5-labeled secondary antibody at 37°C for 60 min. The spheres were then washed 3 times with PBS and the nuclei were counterstained with hoechst33342 for 5 min. Representative images were then acquired by fluorescence microscopy (BX53; Olympus, Tokyo, Japan).

### Immunohistochemistry staining

Spinal cord tissues were fixed in 10% formalin, embedded in paraffin, and then sectioned. Tissue sections were dewaxed in xylene and rehydrated in a series of ethanol concentrations. Immunohistochemical staining was performed according to the manufacturer’s protocols (Boster, Wuhan, China). To the stain spinal cord tissues, sections were incubated with various primary antibodies at 4°C overnight, including anti-Gap-43(1:100, CST, United States), anti-NF200 (Boster, China), anti-SYP (1:100, Abcam, United States), and anti-PSD95 (Boster, China). The following morning, the sections were rinsed in PBS and then incubated with biotinylated polyclonal secondary antibodies. Finally, the sections were washed and incubated with horseradish peroxidase-labeled streptavidin. Sections were then developed with diaminobenzidine and counterstained with hematoxylin. Representative images were acquired using a digital microscope (Nikon, Tokyo, Japan).

### Enzyme-linked immunosorbent assay

On day 7 post-surgery, we collected serum samples from all rats in each group. Subsequently, the expression levels of tumor necrosis factor alpha (TNF-α) and interleukin-1β (IL-1β) were detected by enzyme-linked immunosorbent assay (ELISA) kits (MEIMIAN, Nanjing, China) in accordance with the manufacturer’s instructions.

### Western blot analysis

Cells were lysed in radio-immunoprecipitation assay (RIPA) lysis buffer to extract total protein for western blotting. Subsequently, sodium dodecyl sulfate-polyacrylamide gel electrophoresis (SDS-PAGE) was performed and then the protein samples were transferred to polyvinylidene fluoride (PVDF) membranes. The membranes were incubated overnight with a range of primary antibodies, including ERK (1:1000, CST, United States), p-ERK (1:1000, CST, United States), Gap-43(1:1000, CST, United States), SYP (1:1000, Abcam, United States), CD81 (1:1000, Abcam, United States), Alix (1:1000, Abcam, United States), HSP70 (1:1000, CST, United States), and β-actin (1:1000, Bioworld, United States). The following morning, the membranes were rinsed 3 times in TBS/T and incubated with the horseradish peroxidase-linked secondary antibodies for 1 h at 37°C. Subsequently, immunoreactive bands were visualized by enhanced chemiluminescence and quantified by Image J software (United States). β-actin was used as the internal standard.

### Statistical analysis

All experiments were conducted at least three times. Statistical analyses were performed using GraphPad Prism 8.0 (GraphPad Software, San Diego, CA, United States). All data are presented as mean ± standard deviation (SD). Data analysis relating to BBB scores were conducted by two-way analysis of variance with repeated measures. Other data were analyzed using a one-way analysis of variance and a Bonferroni post-test. *p* < 0.05 was considered statistically significant.

## Results

### Characterization and tracing of hucMSC-sEVs

**FIGURE 1:**
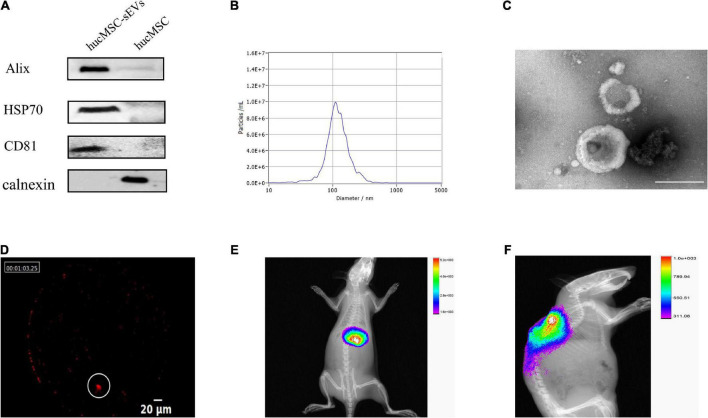
Characterization and tracing of hucMSC-sEVs. **(A)** Western blot analysis of surface markers for hucMSC-sEVs. **(B)** Particle size distribution and concentration measured by NTA. **(C)** Representative image of hucMSC-sEVs by TEM. Scale bars, 200 nm. **(D)** Distribution of Dir-labeled hucMSC-sEVs at the original site of injury 24 h after intravascular transplantation. Scale bars, 20 μm. **(E)** Distribution of Dir-labeled hucMSC-sEVs 24 h after intravascular transplantation *in vivo*. **(F)** Distribution of Dir-labeled hucMSC-sEVs 7 days after intralesional injection *in vivo*.

We successfully isolated sEVs from hucMSCs and identified their characteristics by performing various experiments. Western blotting showed that hucMSC-sEVs expressed typical markers such as CD81, Alix, and HSP70, but not calnexin when compared to hucMSCs; furthermore, these proteins were much more abundant in sEVs than that in cell lysates (Figure 1A). NTA indicated that the diameters of the hucMSC-sEVs ranged from 30 to 500 nm, with a peak size distribution of 110 nm (Figure 1B). The morphology of the sEVs was investigated by transmission electron microscopy (TEM); the hucMSC-sEVs exhibited a characteristic saucer-like shape (Figure 1C). To evaluate the final distribution of hucMSC-sEVs after *in vivo* administration, we incubated hucMSC-sEVs with DiR and performed intravascular application of hucMSC-sEVs by a tail vein of the rat. We found that only a few hucMSC-sEVs were detected at the injury site in the spinal cord (Figure 1D); most of the hucMSC-sEVs had accumulated in the liver when tested 24 h after tail vein injection of DiR-hucMSC-sEVs (Figure 1E). However, most of the hucMSC-sEVs were always concentrated at the site of SCI for 7 days after intralesional injection (Figure 1F). Therefore, the intralesional application of hucMSC-sEVs was performed for all subsequent experiments.

### hucMSC-sEVs promoted functional recovery and attenuated inflammation after spinal cord injury

**FIGURE 2:**
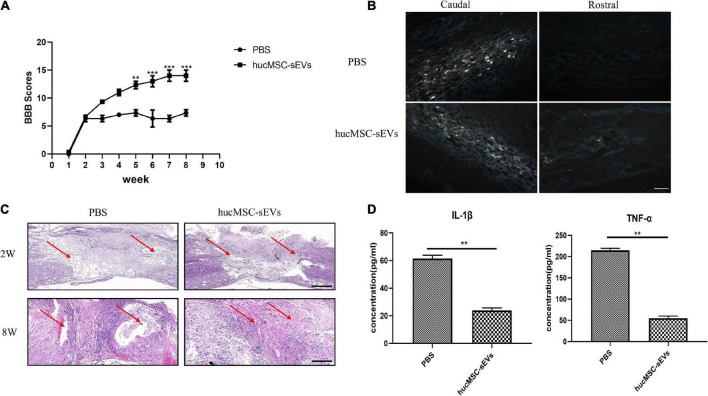
hucMSC-sEVs promoted the functional recovery and attenuated inflammation after SCI. **(A)** BBB scores for rats in the PBS and hucMSC-sEVs treated groups. **(B)** Representative fluorescent-gold retrograde staining of transverse spinal cord sections 8 weeks post-injury. Scale bars, 100 μm. **(C)** H&E staining of spinal cord at 2 and 8 weeks post-surgery. Scale bars, 200 μm. **(D)** Expression levels of inflammatory cytokines (IL-1β and TNF-α) in different groups. The data are presented as means ± SD; *n* = 3; **p < 0.01, ***p < 0.001 vs. PBS group.

To further evaluate whether hucMSC-sEVs exerted neuroprotective effects *in vivo*, we established a rat model of SCI. Firstly, we used the BBB score test to evaluate the recovery of motor function. We found that the BBB scores for the hucMSC-sEVs group were significantly higher than those in the PBS group from the 5th week post-surgery. This indicated that the hucMSC-sEVs had promoted functional recovery after SCI (Figure 2A). Fluorescent-gold retrograde staining of transverse sections of the spinal cord further revealed an abundance of positive neurons at the caudal end of the transverse spinal cord in both groups; there were no positive neurons at the rostral end of the spinal cord in injured rats and several positive neurons at the rostral end of the spinal cord in the hucMSC-sEVs group, thus indicating that the injured nerve fibers were undergoing reconstruction (Figure 2B). The extent of the injured spinal cord was one of the key elements that determined the prognosis of SCI. Hematoxylin–eosin (HE) staining of spinal cord tissue 2 weeks after injury showed that the degradation range of spinal cord tissue was much smaller in the hucMSC-sEVs group than that in the control group, thus indicating the protective potential of the hucMSC-sEVs. Furthermore, H&E staining of spinal cord tissue 8 weeks after injury revealed scarring and that the cavity was larger; a cavity was observed in the control group but not in the hucMSC-sEVs group, thus demonstrating that the hucMSC-sEVs had inhibited the activation of astrocytes (Figure 2C). We also analyzed the expression levels of TNF-α and IL-1β in the serum as an indicator of inflammatory response in the rat models. ELISA results showed that the expression levels of these two inflammatory factors were significantly decreased in the hucMSC-sEVs group (Figure 2D). Collectively, these results indicated that hucMSC-sEVs promoted the repair of SCI and attenuated inflammation after SCI.

### hucMSC-sEVs promoted neurogenesis at the site of spinal cord injury

**FIGURE 3:**
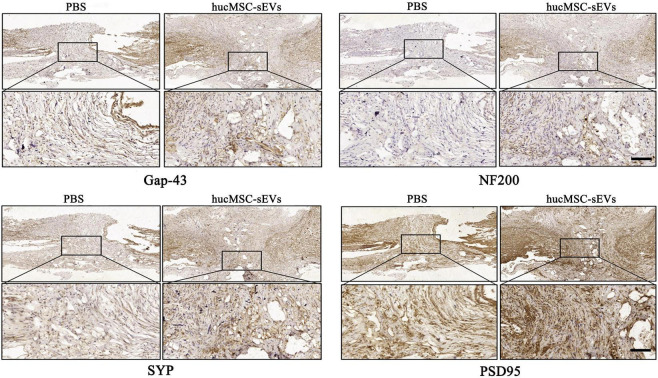
hucMSC-sEVs promoted neurogenesis at the injury site. Immunohistochemical staining of different neural markers at the site of injury in the spinal cord 8 weeks after injury. Scale bars, 100 μm.

Next, we evaluated the efficacy of hucMSC-sEVs with regards to the maturation phenotypes of generating neurons at 8 weeks post-surgery. Immunohistochemical staining showed that the expression levels of both the regenerative neuron marker growth associated protein 43 (Gap-43) and the mature neuron marker neurofilament protein 200 (NF200) in the original lesion site were significantly higher in the hucMSC-sEVs group than in the PBS group. Moreover, the expression levels of both presynaptic and postsynaptic structural marker proteins synaptophysin (SYP) and postsynaptic density protein-95 (PSD95) were also significantly higher in the hucMSC-sEVs group than in the PBS group (Figure 3); these are important indicators reflecting the level of synaptic reconstruction. Collectively, these data suggested that hucMSC-sEVs enhanced neurogenesis at the lesion site in the transverse spinal cord *in vivo*.

### The effect of hucMSC-sEVs on neural stem cell migration and nerve fiber abduction

**FIGURE 4:**
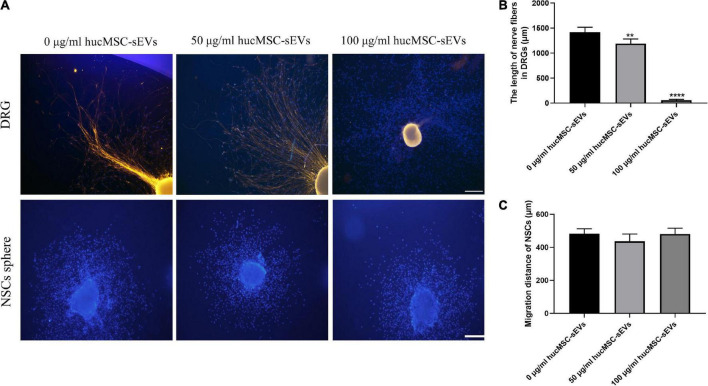
Effects of hucMSC-sEVs on the migration of NSCs and the outgrowth of DRGs. **(A)** Representative immunofluorescence images of axonal marker β-III-tubulin staining in nerve fibers from DRGs and the radial migration test of NSCs from neural spheres *in vitro*. Scale bars, 100 μm. **(B)** The quantitative analysis of the length of nerve fibers in DRGs. **(C)** The quantitative analysis of the migration distance of NSCs. The data are presented as means ± SD; *n* = 3; **p < 0.01,****p < 0.0001 vs. 0 μg/mL hucMSC-sEVs group.

Furthermore, we investigated the effect of hucMSC-sEVs on NSC migration and nerve fiber abduction. First, we examined the potential role of hucMSC-sEVs on the outgrowth of fibers from neurons by DRGs. As shown in Figures 4A,B, in DRG, the blue fluorescence represents the nucleus and the yellow fluorescence represents the nerve fibers, we found that the length of nerve fibers was shorter in the 50 μg/ml hucMSC-sEVs group than those in the 0 μg/ml hucMSC-sEVs group and that nerve fibers were almost invisible in the DRGs treated with 100 μg/ml hucMSC-sEVs. These results illustrated that hucMSC-sEVs had no positive effects on promoting the regrowth of nerve fibers through the transected spinal cord and restoring conduction in the injured spinal cord. Subsequently, we investigated the potential effects of hucMSC-sEVs to induce endogenous NSCs to migrate into the lesion area by detecting radial migration from a neural sphere *in vitro*. We found that there was no significant difference between groups in terms of the number of migrated neural stem cells and the furthest distance by which the NSCs migrated out from the spheres (Figures 4A,C).

### hucMSC-sEVs activated neural stem cells *via* the ERK1/2 pathway

**FIGURE 5:**
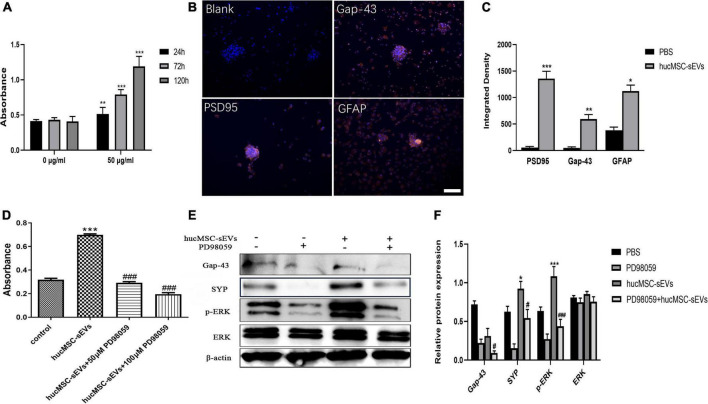
hucMSC-sEVs promoted the proliferation and differentiation of NSCs through the ERK1/2 pathway. **(A)** The cell viability of NSCs treated with 0 and 50 μg/mL hucMSC-sEVs was detected by MTS assays. **(B)** Representative immunofluorescence images of Gap-43, PSD-95, and GFAP staining. Scale bars, 100 μm. **(C)** The quantitative analysis of integrated density of Gap-43, PSD-95, and GFAP in different groups. **(D)** The cell viability of NSCs treated with 50 μg/mL hucMSC-sEVs and PD98059 was detected by MTS assays. **(E)** Representative western blot images showing the protein levels of Gap-43, SYP, ERK, p-ERK. **(F)** Quantification of protein expression. β-actin served as a loading control. The data are presented as means ± SD. *n* = 6 **(A)**; *n* = 4 **(D)**; *n* = 3 **(C,F)**; **p < 0.01,***p < 0.001 vs. 0 μg/mL group; *p < 0.05, **p < 0.01, ***p < 0.001 vs. PBS group; ***p < 0.001 vs. control group, ^###^*p* < 0.001 vs. hucMSC-sEVs group; ^#^*p* < 0.05, ^###^*p* < 0.001 vs. hucMSC-sEVs group, *p < 0.05, ***p < 0.001 vs. PBS group.

To investigate the effects of hucMSC-sEVs on the proliferation and neural differentiation of NSCs, we conducted MTS assays to detect cell viability and performed immunofluorescence staining for neural markers. As shown in Figure 5A, MTS assays demonstrated that the cell viability of NSCs was significantly enhanced after treatment with hucMSC-sEVs. Moreover, immunofluorescence staining showed that the expression levels of the neural markers Gap-43, synaptic structural marker PSD95, and astrocytes marker GFAP were significantly higher in neural stem cell spheres after treatment with hucMSC-sEVs for 1 week (Figures 5B,C), thus indicating that hucMSC-sEVs can promote the differentiation of NSCs into neurons and glial cells. Subsequently, to investigate the molecular mechanisms by which hucMSC-sEVs treatment promotes the proliferation and neural differentiation of NSCs, we investigated the status of the ERK pathway. As shown in Figure 5D, MTS assays showed that the cell viability of NSCs was dramatically increased after incubation with hucMSC-sEVs when compared with the control group while these positive effects were blocked in the presence of an ERK1/2 pathway inhibitor (PD98059). In addition, the western blotting analysis showed that the expression levels of p-ERK1/2 and the neural markers (SYP) were markedly higher in the hucMSC-sEVs group that received hucMSC-sEVs treatment for 48 h; however, the ERK1/2 pathway inhibitor (PD98059) down-regulated the expression levels of these proteins (Figures 5E,F). Collectively, these data suggested that hucMSC-sEVs promoted the proliferation and neural differentiation of NSCs *via* the ERK1/2 signaling pathway.

## Discussion

Spinal cord injury causes severe neurological dysfunction, including paralysis, neurological pain, and gatism, and represents a serious and devastating disease of the central nervous system that is associated with high rates of disability and mortality (Rivers et al., 2018). Thus far, many treatment methods have been used to treat SCI; however, patients with SCI have a generally poor prognosis. In recent years, stem cell-based transplantation treatment strategy has become a potential strategy with which to cure SCI. Previous studies have shown that MSCs have a therapeutic effect on SCI and found that MSCs improved functional recovery, attenuated the formation of glial scars, and promoted the activation of M2 macrophages at the original injury site (Park et al., 2018; Sun et al., 2018). However, the tumor formation potential of grafted stem cells has become a serious obstacle to the application of stem cells in the area of tissue regeneration (Choumerianou et al., 2008). A growing body of evidence now demonstrates that MSCs exert their protective effects, at least in part, through the sEVs produced by paracrine effects. Thus, an increasing number of investigators regard the transplantation of sEVs as a potential alternative to stem cell transplantation. Emerging evidence has proven that sEVs derived from stem cells can exert therapeutic effects for tissue regeneration as a carrier for proteins, RNAs, and DNA (Han et al., 2021; Kang and Guo, 2022). However, there is a lack of direct experimental evidence to show that sEVs derived from MSCs can promote neurological function after SCI; furthermore, the mechanisms involved have yet to be elucidated.

In the present study, we investigated the therapeutic effects of hucMSC-sEVs in a rat model of SCI and explored the underlying molecular mechanisms. First, we successfully isolated and characterized hucMSC-sEVs. We also successfully generated a rat model of SCI at the seventh level of the thoracic vertebra for *in vivo* studies; using these animals, we injected hucMSC-sEVs directly into the lesion site for treatment. Then, we used a small animal *in vivo* imaging system to detect the presence of hucMSC-sEVs and found that sEVs were concentrated at the site of injury for at least 1 week. Spinal cords collected 2 weeks after surgery showed that the extent of the injured spinal cord was smaller in rats from the hucMSC-sEVs treatment group than in the non-treated rats, thus indicating that hucMSC-sEVs exerted protective effects on SCI. H&E staining of the spinal cord 8 weeks after surgery further showed that the cavity was much smaller in the hucMSC-sEVs group. These results illustrated that hucMSC-sEVs reduced the degradation of the damaged spinal cord and inhibited glial scars/cavity formation; both of these aspects are known to exert protective effects on SCI (Wang et al., 2021). The BBB scoring method was used to evaluate the motor function of the hind limbs, thus reflecting the recovery level of spinal cord function. We found that hucMSC-sEVs enhanced functional recovery of the damaged spinal cord. Collectively, these results suggested that hucMSC-sEVs effectively improved the symptoms of SCI. In this respect, our experimental results are consistent with previous studies (Huang et al., 2021; Romanelli et al., 2021). Next, we explored the underlying mechanism from a novel perspective.

Functional recovery depends on the regeneration of axonal fibers through the lesion site and the reconstruction of the neural circuit (Schmued and Fallon, 1986). Thus, we detected regenerative nerves at the site of nerve injury by fluorescent-gold staining. We found that reconstruction of the neural circuit was better after hucMSC-sEVs treatment, thus providing direct histological evidence to explain the functional recovery of the spinal cord. SCI is a complex pathophysiological process in which inflammation plays a key role. Inflammatory reactions, including the release of inflammatory cytokines and the infiltration of inflammatory cells, can cause the apoptosis and necrosis of neurons and glial cells, thus hindering the repair process of SCI (Dutta et al., 2021). In our research, we demonstrated that hucMSC-sEVs inhibited the release of pro-inflammatory cytokines, including TNF-α and IL-1β; this effect is beneficial in terms of both microenvironment regulation and the repair of SCI.

Furthermore, the recovery of motor function mainly depends on the regeneration of neurons and the smooth conduction of upstream and downstream signals at the injury site (Hilton et al., 2017). There are many nerve fibers responsible for upstream and downstream signal transmission and the synapses required to connect nerve fibers in the white matter of the spinal cord. Intact synapses can ensure the sequential conduction of electrical signals between neurons (Filli and Schwab, 2015). In our study, immunochemical staining showed that the expression levels of neuron markers (Gap-43 and NF-200) were higher in the hucMSC-sEVs group. Furthermore, the expression levels of synapse markers (SYP and PSD-95) increased following the intralesional administration of hucMSC-sEVs, thus reflecting the structural integrity and function of the synapses. These findings indicated the regeneration of neurons and synapses at the injury site; these effects are highly beneficial to the recovery of the SCI.

DRGs, aggregates of the neuronal cell bodies, are a classical model with which to investigate the outgrowth of axonal fibers *in vitro* (Vernon and Swanson, 2017). In this study, we detected the migration of axonal fibers from DRGs by the immunofluorescence staining of β-III-tubulin. Results suggested that hucMSC-sEVs do not possess the ability to promote nerve abduction, but directly inhibited the outgrowth of nerve fibers. Previous studies have shown that stem cells can promote the growth and extension of nerve axons (Merianda et al., 2017; Bucan et al., 2019); however, our present results revealed that mesenchymal stem cells derived sEVs inhibited neuronal axon abduction; this could reduce the repair of SCI, at least in part. However, this partial inhibitory effect does not fully represent the overall effect of hucMSC-sEVs on spinal cord transection injury. And, this also demonstrated that the effects of hucMSC-sEVs on the reconstruction of the neural circuit did not involve the promotion of axon extension.

Combined with the results arising from fluorescent-gold retrograde staining, immunohistochemical staining results revealed positivity for neuron and synapse markers in the lesion site; we believe that this immunoreactivity was present in the injury site because of the presence of regenerated neurons. These neurons and synapses must have been derived from NSCs due to the low plasticity of neurons *in vivo*. Therefore, to further investigate the specific molecular mechanisms underlying the neuroprotective effects of hucMSC-sEVs on SCI, we studied the effect of hucMSC-sEVs on NSCs *in vitro.* NSCs possess the potential to differentiate into neurons, astrocytes, and oligodendrocytes; these have strong self-renewal ability and neurotrophic effects (Clarke et al., 2000). The astrocytic differentiation of NSCs leads to glial activation and cavity formation in the damaged spinal cord; these effects are deleterious with regard to the repair process in SCI (Schizas et al., 2018). However, differentiation into neurons and glial cells is one of the main mechanisms for promoting nerve injury repair (Abematsu et al., 2010). In our present study, we explored the potential effects of hucMSC-sEVs to induce NSCs to migrate into lesions by detecting radial migration *in vitro*. We found that hucMSC-sEVs did not improve the migration ability of NSCs; this means that hucMSC-sEVs did not induce the migration of NSCs from the surrounding spinal cord tissue to the injury site to repair SCI. Subsequently, we further investigated the effects of hucMSC-sEVs on the proliferation and neural differentiation of NSCs. Our results demonstrated that hucMSC-sEVs could promote proliferation and the expression levels of the neural markers Gap-43, synaptic structural marker PSD95 and astrocytes marker GFAP; these were significantly increased in NSCs after treatment with hucMSC-sEVs, thus indicating that the neural differentiation of NSCs was enhanced by hucMSC-sEVs. These findings demonstrated that hucMSC-sEVs promote NSCs to proliferate and differentiate.

It has been reported that activation of the ERK1/2 pathway is essential for the proliferation and differentiation of stem cells (Ge et al., 2019; Liu et al., 2019). To further investigate the specific molecular mechanisms underlying the ability of hucMSC-sEVs to induce NSCs to proliferate and differentiate, we investigated the ERK1/2 pathway. We found that after NSCs were treated with hucMSC-sEVs, the expression levels of the p-ERK1/2 were increased, while total ERK1/2 remained unchanged, and that the ERK1/2 inhibitor PD98059 prevented the phosphorylation of ERK1/2, thus blocking the proliferation of NSCs. Moreover, the expression levels of differentiation markers of NSCs, Gap-43, and SYP, were also inhibited by PD98059. These findings suggest that hucMSC-sEVs promote the proliferation and neural differentiation of NSCs *via* the ERK1/2 signaling pathway.

The findings of this study are limited by the fact that we did not explore the downstream molecular mechanisms controlling proliferation and differentiation following activation of the ERK1/2 pathway in NSCs. The exact mechanism of action of sEVs derived from hucMSC in the promotion of proliferation and neural differentiation of NSCs after SCI will be explored in our future studies.

## Conclusion

In conclusion, the present study demonstrated that hucMSC-sEVs can effectively enhance functional recovery, reduce tissue damage, attenuate the inflammatory reaction, and promote NSCs to proliferate and differentiate after SCI by activating the ERK1/2 signaling pathway. These findings may provide a new molecular mechanism for understanding the neuroprotective effects of hucMSC-sEVs and highlight the promising potential of sEVs for clinical application in SCI treatment.

## Data availability statement

The original contributions presented in this study are included in the article/supplementary material, further inquiries can be directed to the corresponding authors.

## Ethics statement

This study was approved by the Medical Ethics Committee and Ethics Committee for Experimental Animals of Jiangsu University (2020161).

## Author contributions

XH, XZhai, WX, XZhou, QF, and HQ designed the study. XH, ZL, XZhou, and QJ contributed to performing the experiments. XH and ZL contributed to performing the data analysis and drafting the manuscript. XZhai, QF, and HQ reviewed, edited, and finalized the manuscript. All authors contributed to the article and approved the submitted version.
